# Supplementary material for: Are carbon emissions trading and green financial instruments synergistic? -Comprehensive quantitative research based on content analysis
Source: PLoS One. 2024 Mar 7;19(3):e0298601. doi: 10.1371/journal.pone.0298601 (PMC10919601; doi:10.1371/journal.pone.0298601)
Supplement: S1 Data — (ZIP) [file pone.0298601.s001.zip › Data/地级市Z_F工作报告.zip!/╜¡╦╒/╬▐╬2╩╨/└┤╫╘╣1⁄2╓┌║┼í╛┬φ┐╦╩2╛▌═°í┐_2008.docx]

各位代表：

　　本届政府2003年1月就职，任期届满。现在，我代表无锡市人民政府，向市第十四届人民代表大会第一次会议报告过去五年的工作，对今后五年及2008年政府工作提出建议，请予审议，并请市政协委员和其他列席人员提出意见。

　　过去五年政府工作的回顾

　　刚刚过去的2007年，是无锡历史上重要而特殊的一年。市政府坚持以邓小平理论和“三个代表”重要思想为指导，牢固树立和落实科学发展观，在中共无锡市委领导下，紧紧依靠全市人民，胜利完成了市十三届人大五次会议确定的各项目标任务。预计，实现地区生产总值3858亿元，同比增长15.3％；财政总收入706.9亿元，同口径增长37.4％，其中一般预算收入300.6亿元，同口径增长36.1％；社会消费品零售总额增长17.8％；全社会固定资产投资增长12.5％；进出口总额增长27.6％，其中出口增长30.6％；城市居民人均可支配收入、农民人均纯收入分别增长13.8％和11％；金融机构各项存贷款余额分别达到4411亿元和3246亿元，其中居民储蓄存款余额1730亿元；城镇登记失业率控制在3.3％以内；国家历史文化名城创建成功，社会事业全面发展，各项工作都取得了新的进步。

过去的五年，是承上启下的重要发展时期，我市胜利完成了“十五”计划，“十一五”规划实现了良好起步，在全省率先基本建成全面小康社会，并向率先基本实现现代化目标顺利迈进。

　　(一)经济结构调整成效显著，区域竞争力全面提升

　　综合实力大幅增强：全市地区生产总值由2002年的1534亿元提高到2007年的3858亿元，年均增长15.7％，实现翻番有余。五年累计实现财政总收入超过2266亿元，年均增长28.6％，其中一般预算收入超过942亿元，年均增长30.6％。全市规模以上工业总产值由2445亿元提高到9000亿元左右。无锡进入全国综合竞争力十强城市行列。产业结构明显优化。三次产业比重调整为1.4：58.5：40.1。高新技术产业发展迅猛，传统产业加快提升。高新技术产业增加值占规模以上工业的比重由26％提高到38.2％左右。集成电路、液晶显示、光伏等优势产业集群逐步发展壮大。五年新增中国名牌产品30个，中国驰名商标16件，国家免检产品历个，阳光精纺呢绒成为全省首个中国世界名牌，无锡跻身中国品牌经济城市行列。

　　新兴服务业和公共服务业发展迅速，服务经济比重提升。社会消费品零售总额由543.6亿元提高到1130亿元左右，人均消费水平居全国城市前茅。旅游业综合实力进入全国十强城市行列。国际招商城、新世界国际纺织服装城、红星美凯龙等一批大型现代化市场蓬勃发展，全市百亿元市场增加到7家，总数占全省一半。现代高效农业规模化发展。到2007年，全市高效农业面积累计突破5万公顷，占全市耕地面积的38％。“一村一品、一村一企”发展战略加快推进，农业品牌建设成果丰硕，省级以上名牌农产品增加到25个，建成国家级农业标准化示范区1个，阳山水蜜桃获中国名牌农产品称号，隆元大米被评为中国名牌产品。节能减排强力推进。综合运用结构调整、源头控制、能源计量和末端治理等多种措施，推广清洁生产，发展循环经济，严格落实重点地区、重点行业、重点企业能耗管理和污染治理措施。预计2007年，万元规模以上工业增加值能耗下降6％；圭要污染物化学需氧量和二氧化硫排放总量有望实现在2005年基础上削减8％的目标。

　　(二)城市化水平稳步攀升，城乡面貌焕然一新

　　功能布局调整优化。强化规划龙头作用，城市总体规划、控制性详细规划及其他专项规划修编完成，以总体规划为核心的规划体系基本建立，城乡规划实现全覆盖。城市发展空间拓展，太湖新城、江阴临港新城、宜兴环科新城和城市重点片区建设改造加快推进。

　　蠡湖新城突出休闲区功能定位，沿湖36公里岸线及十八湾沿湖地区环境综合整治全面完成，建成全省最大的开放式景区，成为山水城市核心标志。全市城市化水平由2002年的59.1％提升到2007年的67.3％。基础设施更加完善。以综合交通体系建设为重点，全面拉开城市框架，两轮“城市建设行动纲要”圆满完成。市区快速路网系统基本构成，市域高等级公路网初步成型，高速公路密度居全省前列。五年来，新拓建高速公路200公里、国省干线公路292公里，新建农村道路1550公里，市区累计建成城市道路205公里。全市自来水日供应能力超过220万吨，市区长江引水一期工程即将完工。

　　按“百年一遇”标准设防的城市防洪工程体系全面建成。500千伏梅里、惠泉等重点输变电工程建成投运。2004年无锡机场开通民航，通航香港、澳门地区及内地20个主要城市，2007年新航站楼竣工投用，进出港旅客人数近136万人次。预计全社会港口吞吐量由2002年的2000多万吨增长到2007年的1.5亿吨，无锡(江阴)港吞吐量跃居长江沿线港口第5位。新农村建设加速推进。第一轮农村五件实事工程全面完成，新五件实事开局良好，现代化新农村建设八项重点工作加快推进。86.4％的乡镇建成了省级以上环境优美乡镇。

　　严格保护基本农田，建设用地实现占补平衡，五年全市土地整理面积累计1.8万公顷。节约集约用地成效突出，累计建成多层标准厂房1500万平方米。全市被征地农民基本生活保障和新型农村合作医疗基本实现全覆盖。环境质量急步提升。把太湖综合整治作为重中之重，大力实施截污调水、清淤控藻、生态修复等工程，水功能区水质达标率逐步提高。五年来，市区污水日处理能力由近31万吨增加到近80万吨，铺设污水管网2100公里，预计城镇污水处理率由56.6％提高到84％。建成2座日处理能力一千吨的生活垃圾焚烧发电厂。大力植树造林，实施惠山青龙山显山透绿工程，积极推进生态湿地和绿色景观建设。五年累计绿化造林3.3万公顷，新增城市绿地3697万平方米。

　　2007年，全市森林覆盖率达到20.5％，空气良好天数占比达到93％，环境质量综合指数82。全市已建成国家卫生城市、国家园林城市和国家环保模范城市群，江阴成为全国首批生态市。

　　(三)开放型经济，陕速增长，全方位开放格局趋于完善

　　外向经济集聚效应凸显。大力引进培育先进产业集群，五年累计到位注．册外资近135亿美元，新批各类外资独立研发中心15个，新增世界五百强企业投资项目51个。海力士大规模集成电路项目成为迄今全省最大的外商独资项目，博世、柯尼卡美能达等一批重大项目建成达产。新区建成全国高新技术标准化示范园区，无锡工业设计园被批准为全国惟一的国家级工业设计园，无锡软件园建成国家火炬计划软件产业基地。对外贸易持续增长，外贸结构不断优化。

　　2007年，预计进出口总额达到500亿美元，比2002年增长4.1倍；其中出口总额280亿美元，增长5.4倍。创立国家级出口品牌6个，列全省第一。无锡成为国家科技兴贸创新基地。开放发展层次提升。

　　服务业开放取得突破，日本瑞穗银行、三菱东京日联银行、沃尔玛购物广场等落户无锡。服务外包发展势头强劲，日本电气、中科院软件研究所、软通动力等成功引进，“中国服务外包示范区——无锡太湖保护区”得到国家四部委联合认定。预计2007年全市服务外包业务总额5亿美元。新批境外投资项目数连续五年保持全省第一，柬埔寨太湖国际经济合作区成为全国首批境外工业园，开发建设取得阶段性成果。城市国际影响扩大。密切与世界各地的经贸、文化交流，与无锡缔结友好关系的城市增加到20个。精心组织太湖博览会、国际工业设计博览会、徐霞客国际旅游节等重大会展活动，积极塑造城市品牌。无锡荣获中国经济活力城市、福布斯内地最佳商业城市等荣誉。

　　(四)改革创新取得重大突破，发展活力明显增强

　　自主创新能力有效提升。把握应用技术自主研发和先进技术自主应用两大重点，加快建设国际先进制造技术中心和区域性创意设计中心，成为全省惟一的国家火炬创新试验城市和国家技术标准试点城市。全面加强产学研合作，大力实施领军型创业人才引进计划，狠抓科技创新型企业培育和科技创业型企业孵化。尚德电力进入世界光伏产业前三强，法尔胜集团被列为国家级创新型企业试点。至2007年，省级以上高新技术企业累计突破1000家，其中国家级高新技术企业97家。全社会研发费用占地区生产总值比重由2002年的0.92％提高到2007年的2％左右。经济体制改革不断深化。全面落实国有企事业单位改革任务，五年累计完成一级企业改革改制599家，完成自收自支经营性事业单位改革改制367家，理顺职工劳动关系近27万人。国有资产监管体制及运行机制逐步完善。村级集体经济股份合作制和社区股份合作社改革全面推开。落实民营企业百强计划，实施“小巨人”工程和“金摇篮”工程。2007年，预计民营经济增加值占地区生产总值比重达58.5％，比2002年提高34.7个百分点。积极引导企业开展资本经营，五年新增上市公司27家，上市公司总数达45家，上市公司数量和募集资金量连续多年在全国地级市中居于首位。行政体制改革加，陡推进。全面实施行政许可制度改革，五年累计削减行政审批事项66％。完善财政管理体制，建立政府购买服务机制，加大对公共服务的保障力度。积极创新社会事业管理体制，推行管办分离改革，社会事业发展机制进一步健全。

　　(五)惠民政策有效落实，人民生活持续改善

　　居民收入稳步提高。推进城乡统筹就业，鼓励群众自主创业，五年累计新增城镇就业63.635人，帮助下岗失业人员再就业27.8万人，促进本地农村劳动力实现就业20.5万人。完善企业职工工资增长机制和欠薪清理机制，企业工资集体协商制覆盖面达85.6％。预计，城市居民人均可支配收入和农民人均纯收入分别由2002年的9988元和5860元提高到2007年的20700元和9860元，年均增长15.7％和11％。社会保障逐步健全。以扩大社会保障覆盖面、加强农村保障制度建设和维护困难群体利益为着力点，基本形成覆盖城乡各类群体的多层次养老、医疗等社会保障制度框架。以公共财政转移支付为特征，施行新型农民基本养老保险制度、城镇无保障老人养老补贴办法和城镇无医疗保障少年儿童、老年居民及其他非从业人员医疗保险制度。目前，城镇社会保险综合参保率达98.1％，农村养老保险综合参保率达87.5％。市区企业退休人员月人均养老金水平由2002年的688元提高到2007年的1197Z。城乡低保实现应保尽保，慈善救助覆盖面不断扩大。

　　实事项目惠及市民。坚持每年办好一批市民普遍关心、群众受益面广的实事项目，切实改善市民生活条件。五年来，经济适用房交付使用237万平方米，农民拆迁安置房建成3500万平方米，符合廉租房政策家庭实现全覆盖。市区改造背街小巷355条，整治老新村312万平方米，市区公交线路扩展到128条，农公班线公交化改造全面完成，镇镇通公交目标顺利实现，70岁以上老人免费乘坐城市公交。

　　(六)社会事业统筹发展，城乡文明程度日益提高

　　公共服务不断完善。加大社会事业投入，五年政府性投入累计达160亿元左右。社会发展综合指数自2003年以来一直位居全省首位。市体育中心、急救中心、疾控中心、老干部活动中心、妇女儿童活动中心及江南大学新校区等全面建成。强化教育资源整合，各类教育协调发展。义务教育阶段免收学杂费、残疾儿童免费入学、进城务工人员子女接受义务教育政策全面落实。五年新增高等院校4所。医疗卫生服务网络进一步健全，市人民医院整合投用，全市社区卫生服务机构实现全覆盖。全民健身服务体系基本建立，竞技体育水平有新的提高，江阴、宜兴、锡山建成全省体育强市(区)。

　　计划生育奖扶政策全面落实。低生育水平保持稳定，人口素质有所改善。社会管理全面加强。改革城市管理体制，深化相对集中行政处罚权改革，深入开展市容环境专项治理，城管创优考核持续位居全省前列。社区基础建设加强，社区服务和管理功能增强。依法治市全面推进，平安创建深入开展，荣获全省社会综合治理先进市和首批社会治安安全市称号。严格安全生产监管，事故起数和死亡人数连续五年下降。整顿和规范市场经济秩序，食品、药品等重点行业稳定有序。建立健全应急管理工作体系，成功应对了非典、禽流感等突发事件。尤其是在处置去年市区部分地区供水危机中，我们把确保群众饮用水安全作为首要目标，围绕改善水源地水质、制水技术攻关、维护社会稳定三大重点，落实一系列应急机制和长效治理措施，在全市上下的共同努力下，用最短的时间恢复了正常供水。

　　文化建设成果显著。弘扬“尚德务实、和谐奋进，’城市精神，以文化力提升城市竞争力。传承优秀历史文化，发展现代先进文化，繁荣艺术精品创作，完善公共文化服务体系。舞剧《红河谷》囊括国家舞台艺术所有最高奖项，锡剧《江南雨》荣获第十届中国戏剧节最高奖——优秀剧目奖。创建全国双拥模范城实现五连冠。审计、统计、农机、史志、档案、侨务、信访、接待、对台、人武、人防、民族宗教、气象、新闻出版、防震减灾、无线电管理等工作取得新成绩，妇女、儿童、老龄、残疾人等事业有了新发展。

　　过去五年中，我们始终坚持以科学发展观为统领，努力把握经济社会发展的阶段性特征和规律性要求，强调工作的连续性、开拓性和操作性，顺势而为，乘势而上，保持了又好又快发展势头。回顾丰富生动的发展实践，有以下体会：

　　——宰宰把握“两个率先”奋斗目标，建设全面小康社会。实现“两个率先”不仅是全市人民团结奋斗的总目标，也是提升无锡发展水平的内在需要。围绕科学发展、和谐发展总要求，我们坚持以经济建设为中心，同时不失时机地加快社会事业发展步伐，更加注重．统筹协调，更加注重社会和谐，顺利实现了第一个“率先”，为第二个“率先”打下了良好的基础。

　　——牢牢把握结构调整工作主线，推进产业现代化进程。步入工业化中后期，加快转变粗放型增长方式，推进产业升级和结构优化，是无锡面临的重大战略任务。我们坚持先进制造业和现代服务业双轮驱动战略，突出高新技术产业和高端服务业主攻方向，大力引进培育，积极嫁接改造，狠抓结构转型，全面促进工业由大变强、服务业能级提升，推动经济大市向经济强市跨越。

　　——牢牢把握城乡一体发展导向，实现区域共同进步。无锡城乡发展水平落差不大，具备统筹发展的基础和条件。我们以城乡规划全覆盖为先导，围绕“七区一体、一体两翼”格局，以城乡布局完善为切入、以基础设施建设为突破、以城市功能提升为重点，大力推进区域城市化进程，中心城市辐射带动作用明显增强，农村综合发展水平明显提升，城乡公共服务差距明显缩小。

　　——牢牢把握环保优先科学理念，增强可持续发展基础。在无锡这样一个资源匮乏、人口密集的地区加快工业化、城市化进程，面临的首要制约是环境的容量、生态的底限。我们贯彻环保优先方针，积极强化环保调控作用，全力落实节能减排措施，破解环境资源瓶颈制约，实现环境质量的逐步改善和提高，努力走上生产发展、生态良好、生活富裕的可持续发展之路。

　　——牢牢把握以人为本核心要求，提高群众生活水平。科学发展的核心是以人为本，发展的根本目的和最终归宿是富裕群众、造福人民。我们努力提高发展的普惠性，把扩大社会就业、提高居民收入、完善社会保障、加强公共服务、强化社会管理作为构建和谐社会的着力点，大力提高社会建设水平，力求让更多人共享改革发展成果，让全体市民过上更加幸福安康的生活。

　　——牢牢把握为人民服务根本宗旨，建社服务型政府。繁重而艰巨的现代化建设任务，要求政府始终保持改革创新、锐意进取的精神，始终保持廉洁高效、求真务实的作风。按照建设人民满意政府的总要求，市政府坚持严格执行法律法规，认真贯彻市人大议案决议，全面加强行政能力建设，规范从政行为，提高行政效能，努力为无锡的发展恪尽职守，尽心尽力。

　　五年征程，五年奋斗，无锡在科学发展的轨道上取得了新的进步与成就。这五年，是无锡改革开放和现代化建设取得重要进展的五年，是我市综合实力大幅提升和市民得到更多实惠的五年，是城市地位和影响力进一步提高的五年。五年的成就，是中央和省正确领导的结果，是全市人民共同奋斗的结果。这里，我代表无锡市人民政府向全市人民及在锡创业务工人员，表示崇高的敬意!向各民主党派、工商联、各人民团体和其他各界人士，向部省在锡单位、驻锡部队和公安干警，表示衷心的感谢！向市内外一切关心与支持无锡改革开放和现代化建设的同志们、朋友们，表示诚挚的谢意!

　　回顾五年发展，我们也清醒地看到，目前我市经济社会发展中还存在一些突出的矛盾和问题：高新技术产业、服务业特别是生产性服务业发展不充分、不成熟，企业自主创新能力整体不强，经济结构现状滞后于发展阶段要求的矛盾较为明显；城镇体系还不完善，中心城市服务功能偏弱，城市规划、建设与管理面临许多新的课题；环境污染的危害仍较突出，节能减排任务繁重，环境保护特别是太湖治理将是一项长期而艰巨的系统工程；城乡居民收入差距有所扩大，区域发展不平衡依然明显，消除城乡制度差异任重道远；文化教育、医疗卫生等社会事业发展还不能满足群众的需求，住房保障、交通出行等仍是百姓关心的热点问题；社会治安、安全生产面临新的挑战，因利益调整而引发的社会矛盾时有显现，维护社会稳定的任务仍较繁重。同时，政府职能转变和作风建设仍有待进一步深化。

　　这些问题，有的是长期积累下来的，有的是体制转轨和结构调整中难以避免的，有的是工作中的缺点和不足造成的，需要我们在今后工作中加以深入研究和切实解决。

　　今后五年的奋斗目标

　　各位代表，今后五年是无锡由重点发展向优化发展转型、全面小康向基本现代化跨越的重要时期。我们必须顺应全市人民过上更加美好生活的新期待，正确把握新阶段新特征，深刻认识面临的新矛盾新挑战，走出一条体现时代特征、符合市情特点的优化发展之路，率先攀登基本现代化新高峰。

　　今后五年，政府工作的指导思想是：全面贯彻党的十七大精神，高举中国特色社会主义伟大旗帜，以邓小平理论、“三个代表”重要思想和科学发展观为指导。紧紧围绕“一当好三争创”目标，始终坚持‘‘富民优先、科教优先、环保优先、节约优先”和“市场导向、创新导向、规划导向、法治导向”方针，全面加强经济、政治、文化、社会建设，确保经济社会全面协调可持续发展。

　　今后五年，政府工作的总体要求是：突出转变发展方式、推进城乡一体发展、建设和谐社会三大主题，加，陕建设“五中心”，努力打造“五名城”，继续保持经济社会发展的良好态势，在全省率先基本实现现代化，努力建成经济繁荣、生活富裕、生态良好、法制健全、社会文明的新无锡。

　　——综合实力明显提升。产业结构更加优化，体制机制更加规范，创新创业环境更加优越，开放水平更加提升。城市综合竞争力继续位居全国城市前列，社会发展指数保持省内领先地位。到2012年，人均地区生产总值达到10万元左右，服务业增加值占地区生产总值比重提高到48％左右，高新技术产业增加值占规模以上工业比重提高到48％左右，全社会研发费用占地区生产总值比重达到2.7％左右。

　　——生态环境显著改善。资源节约型和环境友好型社会建设取得突破性进展，主要污染物排放得到有效控制，基本形成节约能源资源和保护生态环境的产业结构、增长方式、消费模式，建成国家生态城市、生态园林城市、最佳人居环境城市。到2012年，万元地区生产总值能耗下降到0.7吨标准煤左右，环境质量综合指数稳定在90以上。

　　——城乡区域协调发展。城镇空间布局更趋合理，区域城市发展格局基本形成。基础设施体系较为完备，城市管理机制和网络进一步健全，中心城市综合功能显著提升。现代化新农村建设取得重大进展。到2012年，城市化水平达到80％左右。

　　——公共服务全面加强。社会事业发展水平进一步提升，全民受教育程度和创新人才培养水平明显提高，人人享有公共卫生和基本医疗服务，公共文化服务体系和国民体质监测网络覆盖全社会。精神文明和民主法制建设全面加强，市民综合素质进一步提高。社会管理体系更加健全，法治政府建设取得新的成效。人民权益和社会公平正义得到更好保障。

　　——人民生活殷实宽裕。社会就业更加充分，合理有序的收入分配格局基本形成。城乡统筹的社会保险、社会救助、社会福利制度更加完备，人人享有基本生活保障。多层次住房保障体系得到完善。城市居民人均可支配收入和农民人均纯收入年均增长10％以上。

　　为实现上述奋斗目标，在工作中要遵循以下基本原则：

　　一是更加重视扰化发展，进一步提升发展的整体水平。实现重点发展向优化发展全面转型，是新五年发展的根本要求。要优化产业结构，促进制造业向高端延伸，推动服务业在高端突破，全面提升产业层次；优化生态环境，落实环保先导发展的体制机制，加快建设生态文明；优化城市功能，加快构建现代城镇体系，凸显区域城市格局；优化人文素质，培育多元共生、和而不同的开放文化，增强城市的凝聚力和影响力。

　　二是更加重视创新发展，进一步提升城市的核心竞争能力。创新是发展进步的灵魂。要完善市场经济体制，从制度上更好地发挥市场在资源配置中的基础性作用，加快形成统一开放的现代市场体系；强化自主创新，加快完善以企业为主体、需求为导向、产学研紧密结合的技术创新体系，全面提升企业素质，促进全社会科技资源高效配置、综合集成；改革行政管理体制，转变政府职能，健全政府职责体系，强化社会管理与公共服务。

　　三是更加重视统筹发展，进一步提升发展的协调性。推动经济社会更好更大发展，需要处理好各个方面、各个层次的关系。要坚持好宇优先方针，总揽全局，科学规划，努力实现速度质量效益相协调，消费投资出口相协调，一二三次产业相协调，人口资源环境相协调，三级城镇体系相协调，改革发展稳定相协调，逐步形成统筹发展的长效机制。

　　四是更加重视和谐发展，进—步提升全体市民的幸福指数。社会和谐是中国特色社会主义本质属性。要通过发展增加社会物质财富，着力解决群众切身利益问题，努力为市民提供均等化的公共服务、共享化的社会保障和生态化的人居环境，保障市民直接行使民主权利，保障社会公平正义，形成全体市民各尽其能而又和谐相处的局面，创造惠及全体市民的美好生活。

2008年政府工作的主要任务

　　今年是新一届政府履职的第一年，也是全面贯彻落实党的十七大精神的第一年。从经济发展水平和产业综合素质来看，我市总体上已进入工业化后期阶段，面临的发展任务、发展形势和发展环境都有了新的变化。多年改革发展积累了较为雄厚的物质技术基础，我市产业能力、基础设施、体制机制等构成的比较优势依然明显，初步构筑了实现经济社会跨越式发展的较高平台。国际产业结构调整和产业转移纵深推进，为我们加快发展、转型提升提供了良好的机遇。同时也要看到，宏观经济由偏快转向过热的势头尚未缓解。中央着眼于保持经济平稳较快发展势头，进一步实施紧缩性的宏观调控政策，严格控制货币信贷总量和投放节奏，严格控制新开工项目，强化节能减排约束力，给我们推进转型发展创造了机遇，也带来了挑战和压力。国际能源、资源价格大幅上涨，贸易保护、汇率变动等情况复杂交织，无锡经济平稳增长面临的不确定因素增多。我们要全面科学分析形势，善于审时度势，增强机遇意识，坚定发展信念，进一步提高工作的预见性和主动性。

　　综合考虑各方面的因素，今年经济社会发展主要调控指标为：

　　在优化结构、提高效益、降低消耗、保护环境的基础上，地区生产总值同比增长13％以上；万元地区生产总值能耗降低4．5％；主要污染物排放量在2005年基础上累计削减12％；环境质量综合指数稳定达到82以上；地方财政一般预算收入同口径增长15％以上；城市居民人均可支配收入增长13％；农民人均纯收入增长10％；城镇登记失业率控制在3．5％左右。

　　(一)实施创新战略，构筑现代产业体系

　　产业结构决定发展水平和城市综合竞争力。要加快经济结构战略性调整，促进产业优化升级，促进区域生产力合理布局，积极构建可持续发展的现代产业体系。建设创新型城市。坚持一手抓高新技术企业发展壮大，一手抓科技创新企业培育孵化，努力使企业在自主创新中成为投资主体、研发主体和应用主体。强化政府科技投入，加快公共服务平台建设，健全政产学研紧密结合的区域创新体系，推动创新成果与市场和资本加速对接。继续实施海外领军型创业人才引进"530计划”，落实各项优惠政策。全社会研发费用占地区生产总值比重达到2．1％。培育优势产业。立足于雄厚的工业基础，大力推进信息化与工业化融合，促进电子信息、机械装备等主导产业向高端延伸，促进与之密切相关的生产性服务业专业化、规模化发展。大力培育集成电路、光伏、软件、动漫等高新技术产业和高端服务业，力争在创意设计、信息咨询、金融保险、现代物流等领域形成特色优势。以休闲名城建设为统领，加快旅游、商贸等相关产业的联动发展，努力提高城市生活环境品质。提升企业素质。着力优化企业发展环境，切实提高企业人员素质，大力推广卓越绩效管理，有效推动企业履行社会责任。全面实施质量和知识产权立市方针，推进品牌战略、商标战略和标准化建设。发展规模经济，培育一批主业突出、核心竞争力强的大企业大集团。改善内需结构。坚持扩大内需方针，着力提高投资效益，着力使消费逐步成为经济增长的持久动力。力争社会消费品零售总额增长16％，全社会固定资产投资增长14％。优化投资结构，引导扩大服务业和基础设施投入，增加技术改造和技术设备投入。努力创新服务业态，积极引进国际服务品牌，进一步提升市场建设水平，大力发展连锁经营，有效改善集贸市场环境。规范市场经济秩序，强化粮油、肉类等生活必需品市场保供措施，完善食品质量安全监管。居民消费价格指数控制在国家及省调控目标之内。

　　(二)着眼城乡一体，加快区域城市化进程

　　走区域城市化发展之路，是无锡城市化和城市现代化发展的目标取向。要进一步深化城乡规划体系，加快建设开放共享、配套完善的城乡基础设施，扩大中心城市辐射带动半径。统筹城乡布局。

　　按照最适规模、最适密度、最适分工的思路，加快构建布局合理、功能清晰的现代城镇体系。严格落实城乡规划全覆盖，深化生态环境、历史文化遗产和传统风貌保护规划，完善城乡市政基础设施及公共服务设施规划。坚持旧城改造与新城开发相结合，集中力量建设太湖新城、锡东新城等五大新城，进一步提升城市重点片区功能。

推进城市现代化建设。围绕“建设东西通道、连接新老城区、形成快速路网”目标，全面启动第三轮“城市建设行动纲要”，新拓建人民路、永乐路、红星路、吴越路等城市道路。着力培育苏南国际航空港，积极实施高速铁路和城际铁路无锡站及配套项目，推进会议中心、会展中心、文化艺术中心等工程建设。加强供水、电力、燃气、信息、停车场等公共设施建设力度。按三级航道标准，全面实施苏南运河无锡段升级整治工程。全力推进长江引水工程，确保一期供水工程4月底前建成投运，并开工建设二期工程，保障生活、生产用水安全。加大国家历史文化名城保护力度，加强非物质文化遗产保护，实施惠山、清名桥等历史街区保护与开发，加快阖闾城遗址考古发掘和鸿山遗址公园建设。建立健全城市管理网络，加强城管队伍建设，全面实行属地管理责任制。有效协调日常管理和行政执法工作，加快推进数字城管建设，实现城市管理常态化、长效化。加快农村发展。以农业增效、农民增收为目标，推进现代农业综合创新试点，积极探索苏南现代高效农业发展之路。强化土地集约利用和计划管理，合理安排建设用地，积极开展大规模土地整理。加快农村“三集中”工程，大力开展以治乱、治脏、治污为重点的农村环境综合治理，再现江南水乡秀丽景色。

　　(三)深化改革开放，激发全社会创业动力

　　新时期最鲜明的特点是改革开放。要以解放思想为先导，进一步深化综合配套改革，全面提高对外开放水平和城市国际化程度，建立健全促进科学发展、和谐发展的体制机制保障。激发对外开放带动力。加快开放型经济转型升级，建设好国家服务外包示范区，大力吸引海内外研发机构聚集，加速壮大优势产业集群，实现利用外资向产业链高端环节攀升。以培育龙头企业为关键，重点落实“123”计划，大力发展软件及服务外包产业。加强与世界知名认证机构合作，推动服务外包企业开展国际资质认证。加快培育自主出口品牌，扩大高新技术产品出口份额。力争进出口总额增长13％。鼓励有条件的企业继续兴建各类境外企业和特色园区。继续结交一批国际友城，加强对外交往与合作，加快教育、卫生、体育等社会事业开放步伐。激发民营经济爆发力。进一步放宽市场准入，大力营造鼓励创业、支持创业的社会氛围，放手发展民营经济。强化创业服务中心功能，完善流程服务和辅导培训，建立健全会计、公证、融资等中介服务组织。充分发挥创业风险投资基金作用，重点投向处于种子期和起步期的创业企业，大力扶持中小型成长企业。加快培育民间投资市场，积极引导民间资本参与基础设施和社会公共设施建设。激发体制机制原动力。把发展壮大股份制和混合所有制经济放到更加突出的位置，促进多元资本相互融合，增强微观经济发展动力。优化国有经济布局和结构，完善国有资产监督管理体制和运行机制，建立健全国有企业法人治理结构。扩大金融开放，整合金融资源，提高金融市场活力、金融服务水平和抗风险能力，努力建设金融强市。加快多层次融资体系建设，推动各类企业上市融资，进一步壮大“无锡板块”。

　　(四)打谴强势环保，推进生态文明建设

　　生态是发展的基础。要切实把环境保护放到经济社会发展的先导位置，突出源头控制、过程控制、全程控制，促进环境质量持续改善。狠抓节能减排。积极扶持生态型、节能型产业优先发展，严格限制并坚决淘汰环境资源依赖程度大、生产工艺落后的企业，完成“三高两低”和“新五小”企业整治任务，持续打好节能减排攻坚战。万元规模以上工业增加值能耗降低5％。建立环境污染赔偿和资源利用奖惩机制，实行主要污染物总量削减保证金和环境资源区域补偿制度。加速构建循环经济体系，依法实施清洁生产审核。强化环境治理。进一步提高污水处理能力，严格执行大湖地区城镇污水处理厂及重点工业行业主要水污染物排放标准，确保实现太湖治理规划确定的治理目标。强化污水接管措施；切实保证城镇生活污水集中入网。城镇生活污水集中处理率达到85％。狠抓重点污染企业治理，全面完成排污口整治。完善“河长制”管理，确保断面水质达标。

　　切实加强面源污染整治，严格落实保护区种植、养殖限制规定。加快创建国家节水型城市，普及推广节水器具，实施污水再生利用工程。全面治理机动车尾气，积极控制城市扬尘，扩大烟尘控制区覆盖范围，加快城市清洁能源区建设。严格噪声控制和管理，建设宁静城市。加快城乡生活垃圾收集体系建设，形成以焚烧为主的处理方式。完成桃花山垃圾填埋场扩建一期工程和渗滤液预处理工程，开工建设日处理能力两千吨的锡东生活垃圾焚烧发电厂。加强固体废弃物回收利用，推进资源化循环。加大环境监测、监控力度，完善环境应急机制。建设绿色家园。改善和加强大湖、长江和横山水库等饮用水源地保护措施，全面推进太湖一级保护区生态防护林、湖滨生态湿地、水生植物示范工程建设，加快退耕还湖、还林、还湿地，改善水域自然生态环境。加大森林保护和城乡绿化建设力度，积极开展绿色村镇、绿色家园、绿色单位创建，加强太湖风景名胜区保护和管理，提升环境整体水平和景观质量。全年完成造林绿化6700公顷，森林覆盖率达到22％，新增城市绿地600万平方米。

　　(五)加强社会建设，推动社会全面进步

　　完善公共服务、改进社会管理，是构建和谐社会的客观要求。

　　要进一步加大社会事业投入力度，健全社会管理体制机制，最大限度减少不和谐因素。建设教育强市。确保政府对教育的投入，全面实施素质教育，重视学前教育，关心特殊教育，促进教育公平。深化职业教育改革，推进产学合作和实训基地建设。继续扩大高等教育规模，发展远程教育和继续教育，建设全民学习、终身学习的学习型社会。提高全民健康水平。坚持公共医疗卫生的公益性质，强化政府责任和投入，实现医疗卫生事业大发展。健全基本医疗卫生服务网络，推进医疗卫生资源整合，加快发展城乡社区卫生服务，高标准建设社区卫生服务中心，实行低成本收费，方便群众就医，减轻就医负担。施行基本药物制度，提升食品药品质量安全水平。

　　加强疾病预防控制，加快公共卫生应急体系建设。推进国家级全民健身基地及何振梁与奥林匹克陈列馆建设，率先建设体育强市。提升城市文明素质。繁荣哲学社会科学，积极发展文学艺术、新闻出版、广播电视事业，加强网络文化建设与管理。完善公共文化设施，加快建设无锡博物院新馆、中国乡镇企业博物馆等工程。推动文化资源向文化资本转化，加快培育富有竞争力的文化产业。深入开展群众性精神文明创建，大力普及科学知识和法律知识，建立健全社会诚信体系。大力实施人才强市战略，全面提升人口素质。加强国防后备力量建设和双拥工作。承办好第二届世界佛教论坛。强化基层基础建设。深化村民自治和居民自治工作，进一步理顺基层社会管理体制，全面推进社区事务工作站建设，确保基层管理必需的人员和经费投入。加强城乡社区公共服务设施建设，完善社区功能，拓宽与群众生活密切相关的服务内容，推进城乡社区基本公共服务均衡化。

　　(六)着力改善民生，提高群众生活质量

　　改善民生是和谐社会建设的重点，也是政府工作的出发点和落脚点。要以解决人民群众切身利益问题为重点，切实做到以民为本，以民为先，惠民为重。发展和谐劳动关系。深入实施积极的就业政策，建立健全公共就业服务体系，进一步完善面向所有困难群体的就业援助制度。完善收入分配制度，创造条件让更多群众拥有财产性收入。严格执行最低工资标准，加强劳动保障监察执法，维护劳动者权益。力争实现城镇新增就业10万人，在岗职工平均工资增长13％以上。继续增加企业退休人员养老金。完善社会保障制度。以基本养老、基本医疗、最低生活保障为重点，加快完善社会保障体系。继续推进社会保险扩面，加大对参保人数、缴费基数的监察执法和稽核力度。确保企业养老保险扩面12万人，基本医疗、失业、工伤、生育等保险均扩面5万人以上。加快构建覆盖城乡的新型社会救助体系，推动社会救助逐步向普惠型的社会福利过渡。完善临时救助制度，提高对困难群众的及时保障能力。积极发展老龄事业，逐步实现高龄、贫困老人无障碍进入养老机构安度晚年。大力发展慈善事业，引导社会各界参与慈善活动。改善市民生活条件。全力为民办好十件实事。建立多层次住房保障体系，将廉租房保障对象扩大到城市低收入住房困难家庭，加快经济适用房和拆迁安置房建设，多渠道解决困难群众住房保障问题。着力推进城区危旧房、城中村和老新村改造整治。加强房地产市场调控，维护和促进房地产市场持续健康发展。围绕“公交优先、企业优秀、服务优质”目标，加大公共财政投入力度，落实公交发展规划，加快基础设施建设，加强运行监管，提高公交服务水平。维护社会安定有序。建立健全应急管理体系，提高对突发公共事件的预警和处置能力。拓宽和畅通社情民意表达渠道，把群众利益诉求纳入制度化、规范化、法制化轨道。强化企业主体责任，严格安全生产监管。提高防汛抗旱、防震减灾等综合能力。深入开展平安创建活动，健全社会治安防控体系，依法防范和打击违法犯罪活动，维护社会秩序和公共安全。

　　(七)切实转变职能，建设人民满意政府

　　新的形势和任务迫切需要我们加强行政管理体制改革，加快建设服务型政府，不断提高管理经济社会事务的能力和水平。创新行政体制。着力转变职能，理顺关系，提高效能，努力形成权责一致、执行顺畅、监督有力的行政管理体制。健全公共财政体制，不断优化财政支出结构，完善公共服务体系，提高公共服务质量。深化行政审批制度改革，进一步完善行政服务机制。扩大政务公开，发展电子政务，建立健全政府新闻发布制度，保障人民群众的知情权、参与权和监督权。严格依法行政。加强政府法制工作，加快建立权责明确、行为规范的行政执法体制，强化对决策和执行等环节的监督，确保各级行政管理机关严格按照法定权限和程序行使职权、履行职责。提高公务人员法律素养，做到依法决策、依法行政、依法管理。强化作风建设。

　　全面落实廉政建设责任制，以制度建设为重点，规范行政行为，切实做到用制度管人、管事、管钱，从根本上预防和治理腐败。推进学习型机关建设，加强公务员队伍教育、培训和管理。完善考核体系和奖惩机制，切实增强服务基层、服务群众的公仆意识，努力形成“为民、务实、清廉”的良好政风。

　　各位代表，无锡的前景美好而光明，我们的使命光荣而神圣!让我们紧密团结在以胡锦涛同志为总书记的党中央周围，在中共无锡市委的领导下，全面贯彻落实科学发展观，继往开来，坚韧不拔，奋力拼搏，开拓进取，为率先攀登基本现代化新高峰，建设和谐幸福新无锡而努力奋斗!
